# Correction Notice: Single-step Precision Genome Editing in Yeast Using CRISPR-Cas9

**DOI:** 10.21769/BioProtoc.3610

**Published:** 2020-04-05

**Authors:** Azat Akhmetov, Jon M Laurent, Jimmy Gollihar, Elizabeth C Gardner, Riddhiman K Garge, Andrew D Ellington, Aashiq H Kachroo, Edward M Marcotte

**Affiliations:** Center for Systems and Synthetic Biology, Institute for Cellular and Molecular Biology, University of Texas at Austin, Austin, TX, USA; Institute for Systems Genetics, Department of Biochemistry and Molecular Pharmacology, New York University Langone Health, New York, NY, USA; The Department of Biology, Centre for Applied Synthetic Biology, Concordia University, Montreal, QC, Canada; Department of Molecular Biosciences, University of Texas at Austin, Austin, TX, USA

After official publication of our protocol in bio-protocol (https://bio-protocol.org/e2765), we noted some errors in the protocol and wished to correct the protocol. The edits to be performed are as the following:

1. The original Figure 1 does not appropriately indicate the generation of the Cas9 transcription unit. The process of how to create a Cas9 transcription unit with suitable promoters, terminators, and connectors has now been included in the new Figure 1. All the transcription units were made in the pYTK095 background, which has also been edited.
  **Figure 1.**
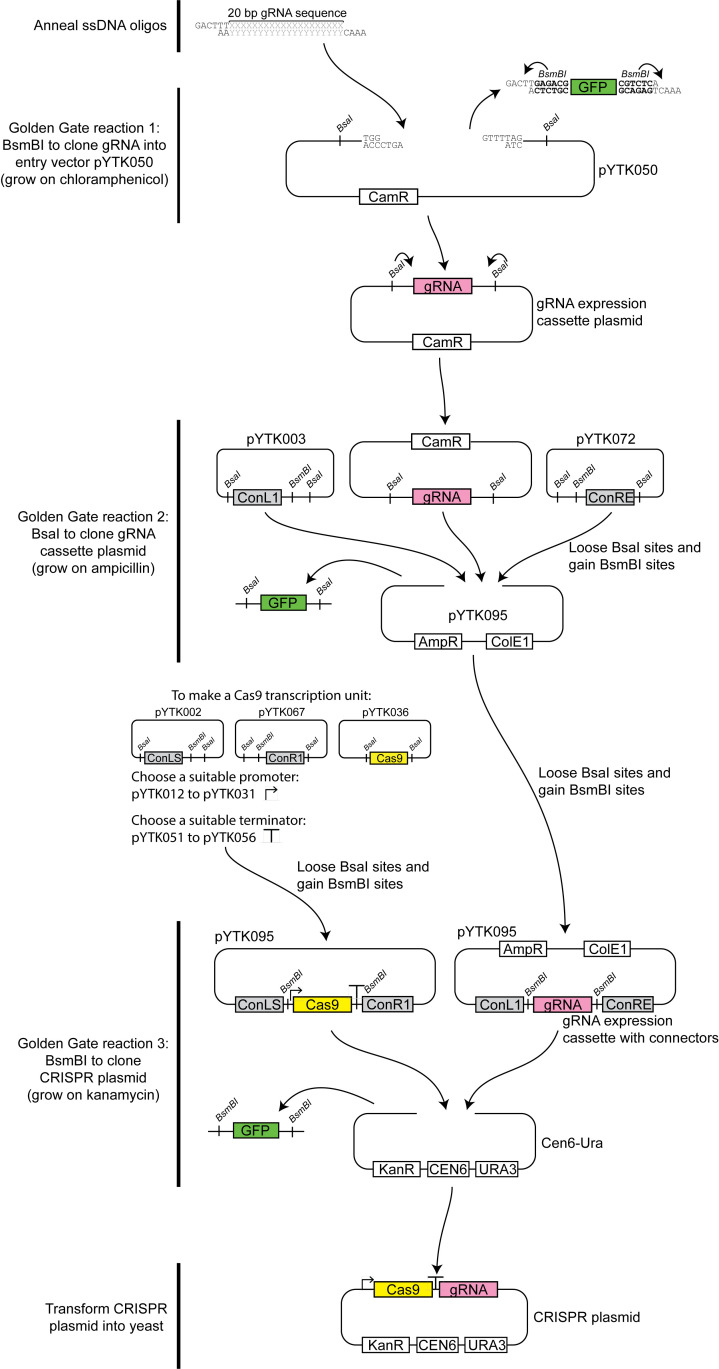
Overview of the CRISPR/Cas9-gRNA expression vector construction process. In the first step Xs and Ys represent the gRNA sequence selected, and BsmBI recognition site is indicated in bold.
2. Table 2 now shows an additional reaction for generating the transcription unit for the Cas9, as shown in Figure 1. The new Table 2 is attached below.
  **Table 2:**
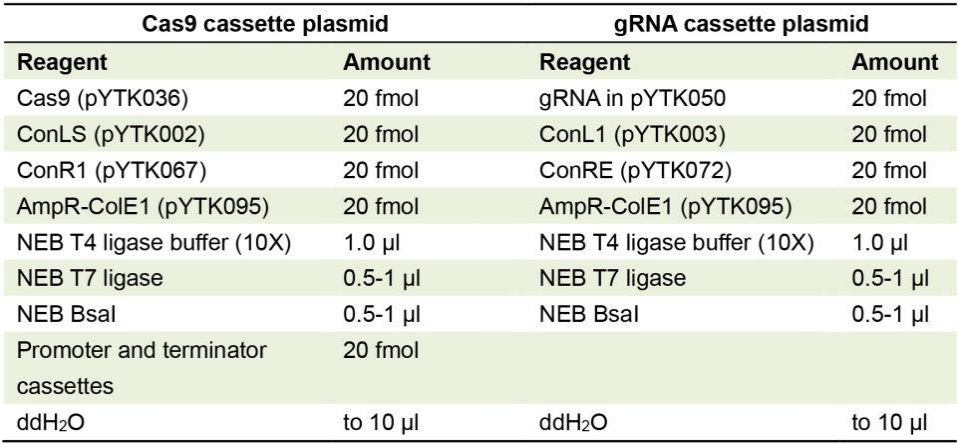
Golden Gate reaction for making Cas9 and gRNA transcription unit/cassette plasmids with appropriate connectors
3. Table 3 has also been edited that includes the title and footnote edits. The edits are the following:
  **Table 3:**
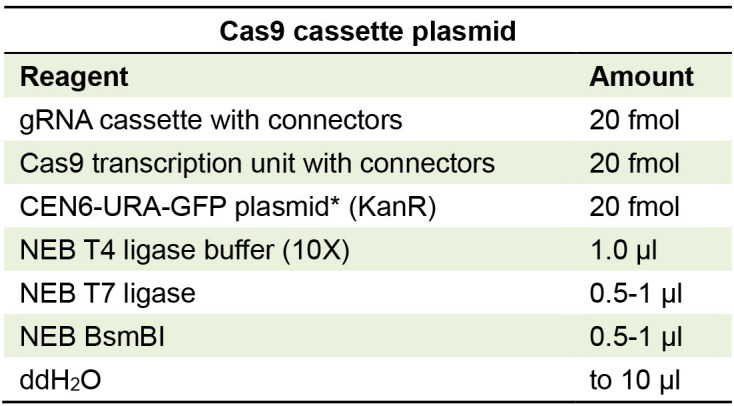
Golden Gate reaction for making Cas9 and gRNA yeast expression vector *Cen6-Ura is constructed by assembling YTK plasmids (008, 047, 073, 074, 081, and 084) using BsaI enzyme and End-ON-Ligation step for the Golden Gate reaction.
  a. The correct cassette plasmids with connectors are indicated in the reagents section.
  b. The volumes of ligase and enzymes have been edited from 0.5 µl to 0.5 - 1 µl.
  c. The Title shows that this plasmid now serves as an expression vector in a yeast cell.
  d. The footnote for the table now indicates the End-On-Ligation step required at the end of the Golden Gate reaction cycle to generate a CEN6-URA-GFP vector.

## 
References



Akhmetov, A., Laurent, J. M., Gollihar, J., Gardner, E. C., Garge, R. K., Ellington, A. D., Kachroo, A. H. and Marcotte, E. M. (2018).  Single-step precision genome editing in yeast using CRISPR-Cas9. 
*Bio-protocol* 8(6): e2765.


